# Frontoparietal functional connectivity moderates the link between time spent on social media and subsequent negative affect in daily life

**DOI:** 10.1038/s41598-023-46040-z

**Published:** 2023-11-22

**Authors:** Yoona Kang, Jeesung Ahn, Danielle Cosme, Laetitia Mwilambwe-Tshilobo, Amanda McGowan, Dale Zhou, Zachary M. Boyd, Mia Jovanova, Ovidia Stanoi, Peter J. Mucha, Kevin N. Ochsner, Dani S. Bassett, David Lydon-Staley, Emily B. Falk

**Affiliations:** 1 Department of Psychology, Rutgers, The State University of New Jersey, Camden, NJ 08102 USA; 2 Annenberg School for Communication, University of Pennsylvania, Philadelphia, PA 19104 USA; 3 Department of Psychology, University of Pennsylvania, Philadelphia, PA 19104 USA; 4 Department of Psychology, Concordia University, Montreal, QC H4B 1R6 Canada; 5 Department of Neuroscience, University of Pennsylvania, Philadelphia, PA 19104 USA; 6 Department of Mathematics, Brigham Young University, Provo, UT 84604 USA; 7 Department of Psychology, Columbia University, New York, NY 10027 USA; 8 Department of Mathematics, Dartmouth College, Hanover, NH 03755 USA; 9 Department of Physics and Astronomy, University of Pennsylvania, Philadelphia, PA 19104 USA; 10 Wharton Marketing Department, University of Pennsylvania, Philadelphia, PA 19104 USA; 11 Wharton Operations, Information and Decisions Department, University of Pennsylvania, Philadelphia, PA 19104 USA

**Keywords:** Emotion, Social neuroscience, Human behaviour, Risk factors

## Abstract

Evidence on the harms and benefits of social media use is mixed, in part because the effects of social media on well-being depend on a variety of individual difference moderators. Here, we explored potential neural moderators of the link between time spent on social media and subsequent negative affect. We specifically focused on the strength of correlation among brain regions within the frontoparietal system, previously associated with the top-down cognitive control of attention and emotion. Participants (N = 54) underwent a resting state functional magnetic resonance imaging scan. Participants then completed 28 days of ecological momentary assessment and answered questions about social media use and negative affect, twice a day. Participants who spent more than their typical amount of time on social media since the previous time point reported feeling more negative at the present moment. This within-person temporal association between social media use and negative affect was mainly driven by individuals with lower resting state functional connectivity within the frontoparietal system. By contrast, time spent on social media did not predict subsequent affect for individuals with higher frontoparietal functional connectivity. Our results highlight the moderating role of individual functional neural connectivity in the relationship between social media and affect.

## Introduction

Over 4.5 billion people around the world use social media, with the average daily user engaging in 2.5 h in 2022^1^. The wild popularity and proliferation of social media have prompted scientific investigation into potential effects of social media use on health^2^. One insight we have gained from the past decade of research is that the question of how social media relate to well-being does not have a single answer. Social media influence people differently, because different people engage with social media contents differently^3^. Consistent with this view, emerging evidence highlights that the relationship between social media and well-being is inconsistent^4–7^. For example, a recent large-scale meta-analysis of 226 studies showed that although social media use was associated with small improvement in social well-being, the benefit also came at the cost of increased negative emotions such as depression and anxiety^8^. Furthermore, some of these effects varied according to a range of moderators, suggesting that the effects of social media may depend on a number of factors that vary across the individual, platform, context, and more^5^. Various candidate moderators have been examined, including demographics, personality traits, types of social media use, and media content^8^. However, little is known as to whether individual differences in intrinsic functional neural architectures may moderate different links between social media use and symptoms of psychopathology or well-being.

Individual differences in the brain systems that support cognitive control, in general, including over one’s emotions, may be a key moderator for the relationship between social media use and affective outcomes. Neurocognitive control circuits support attention to different stimuli^9^ and may guide whether and to which aspects of social media contents one attends, as well as the interpretation and re-interpretation of perceived contents^10^. Resting state functional connectivity, or the synchronous activity between different regions of the brain at rest, offers one way to observe the function of circuits that underlie cognitive control^11^. In particular, the frontoparietal system has been implicated in cognitive control of affective processes^12–14^ in individuals with^15^ and without psychopathology^16^. The frontoparietal system is composed of dorsolateral prefrontal cortex and posterior parietal cortex^9^ and is extensively interconnected with other systems, such as default mode and attention systems^17^, that support distinct and competing psychological processes underlying the regulation of social and affective experiences^18,19^.

In support of the frontoparietal systems’ connection to emotion outcomes, problematic negative emotions have been associated with disrupted resting state functional connectivity within brain systems involved in emotion regulation^20,21^. Specifically, differences in frontoparietal connectivity, including both the hypo- and hyper-connectivity, have been implicated in increased negative affect (vs. other types of emotion more generally) and are a widespread feature of virtually every major mood disorder^22^. Differences in the frontoparietal system, such as hypoconnectivity between its constituent regions^22,23^, have been linked to difficulty regulating responses to emotionally salient information, resulting in emotion dysregulation^24^.

More specifically, how may the functional connectivity within the frontoparietal system relate to the ways people experience social media? By design, social media platforms are replete with information that may affect emotion. Some include potential sources of positive affect such as faces and personal narratives of close others^25^. However, social media can increase a risk of harm to one’s overall mental health^26^ (United States) by increasing negative affect via multiple mechanisms, for example, by serving as a source of negative news contents^27^, online aggression^28^, and other social information that could negatively impact well-being for certain individuals (e.g., individuals who are prone to social comparison^29^). When exposed to emotionally salient information on social media, weaker connectivity among the nodes of the frontoparietal system may signal a reduced ability to regulate affective responses, for example by diverting attention from negative contents or (re)interpreting content in adaptive ways^10^, potentially increasing negative affect following social media engagement.

Compared to the robust link between disrupted frontoparietal functional connectivity and psychopathology^22,23^, the role of resting state frontoparietal functional connectivity in negative affect among non-clinical populations—which may comprise a large portion of the social media users—is less clear^15^. Studies examining how functional connectivity within neural structures relates to the affective experience and difficulties in regulating negative emotions in non-clinical populations could elucidate how complex social stimuli, such as social media contents, may relate to people’s everyday affective experience and well-being more broadly. Examining how frontoparietal connectivity relates to people’s day-to-day affective outcomes can also help test ideas about psychological processes involved in social media effects that might not be accessible via self-reports alone.

The current study examined potential neural moderators of the within-person temporal link between time spent on social media and subsequent affect. We assessed resting state intrinsic connectivity within the frontoparietal system because this system is implicated in the cognitive control of emotion. We specifically focused on negative affect outcomes following social media use, based on previous evidence on potential harms of social media on mental health^26^, as well as the role of frontoparietal functional connectivity in negative affect regulation^22^. We first tested whether lower frontoparietal connectivity would be associated with greater self-reported negative emotions (i.e., depression and anxiety) and difficulties in emotion regulation. Next, using an intensive, longitudinal ecological momentary assessment (EMA) design, we examined the within-person temporal relationship between the amount of time spent on social media and subsequent negative affect. We further explored whether the link between social media use and subsequent negative affect would be moderated by individual differences in resting state frontoparietal functional connectivity. We hypothesized that lower frontoparietal connectivity would be associated with a stronger link between social media use and subsequent negative affect.

## Methods

### Data, code, and protocol availability

Data and analysis scripts are available at https://github.com/cnlab/social_media_brain/. The current study reports a subset of data from a parent study, and the methodological details on participant recruitment, data collection, and functional magnetic resonance imaging (fMRI) data preprocessing have also been described in the study protocol paper^30^ and a study that examined alcohol use behavior^31^ that is not the focus of the current report.

### Participants and procedure

This multisite study recruited students attending two urban universities in the United States of America who belonged to different campus groups at the time of recruitment. Based on the initial online survey responses, 111 participants who met the fMRI eligibility criteria visited the laboratories, completed surveys that assessed their baseline levels of depression and anxiety, completed an fMRI visit, and had usable data. All participants who completed fMRI were invited to an initial round of EMA that did not contain any social media use questions, and hence was not the focus of the current report. About nine months (mean = 307.8 days; median = 280 days; SD = 135.75; range = 85–533) after the fMRI scan, at the start of the coronavirus (COVID-19) pandemic, all participants were once again invited to complete another round of 28-day EMA that included both the social media use and affect measures relevant to the current investigation. In this round, 54 of the participants who completed the baseline fMRI also finished the EMA portion of the study with usable data (M_age_ = 20.35 years; SD_age_ = 1.32; 37 women, 16 men, 1 indicated to be “Other” gender; 26 White, 16 Asian, 2 Black, 3 Latino/a, and 7 Multiracial).

Eligibility criteria for the fMRI visit included standard MRI eligibility criteria (no metal in body, not claustrophobic, not pregnant/nursing, and weighs less than 350 lb due to the scanner weight limit), older than 18 years of age, fluent in English, not currently studying abroad, and having no history of serious medical history, psychiatric hospitalization, or substance abuse. Exclusion criteria as part of a parent study that are unrelated to the current report included having never had alcohol or consuming less than one drink in a typical drinking occasion, and not having at least two friends who drink alcohol, one more than the self and the other less than the self. This study was approved by the University of Pennsylvania Institutional Review Board and the Army Research Office’s Human Research Protection Office. All experiments were performed in accordance with the Declaration of Helsinki and with relevant guidelines/regulations. All participants provided informed consent and were paid for their participation. Online surveys were conducted via Qualtrics, scanner tasks were presented using PsychoPy2^32^, and the EMA prompts and participants’ responses were delivered via the LiveData app (www.lifedatacorp.com).

### Resting state fMRI

#### Data acquisition and preprocessing

Resting-state fMRI (rs-fMRI) and structural images were acquired using 3 Tesla Siemens Prismas with 64-channel head coils at the University of Pennsylvania Center for Functional Neuroimaging and at the Mortimer B. Zuckerman Mind Brain Behavior Institute at Columbia University. The acquisition protocols were identical across sites. For the resting state scan, we collected 300 continuous echo-planar imaging (EPI) functional volumes with the following parameters: voxel size = 3 × 3 × 3 mm; 42 slices; field of view (FOV) = 210 mm; time repetition (TR) = 1000 ms; time echo (TE) = 30 ms; multiband acceleration factor (MBAF) = 3; flip angle = 62. During the resting state scan, participants were instructed to keep their eyes open and focus on a fixation cross for 5 min. A MPRAGE anatomical scan was also collected using the following sequence: voxel size = 0.9 × 0.9 × 1.0 mm; 160 slices; FOV = 240 mm; TR = 1850 ms; TE = 3.91 ms; flip angle = 8. The neuroimaging data were preprocessed using fMRIPrep (Version 20.0.6)^33^ based on Nipype 1.4.2^34,35^. Please see Supplemental Information 1 [SI1] for details of the structural image preprocessing.

The rs-fMRI were preprocessed with the following steps: (1) slice time correction with AFNI 20160207^36^ and (2) motion correction using rigid body translation and rotation with FSL 5.0.9^37^. The functional and structural images were aligned using Freesurfer. We calculated various confounds (e.g., framewise displacement [FD], DVARS, global signal) for each TR. Across the 300 total volumes of the participants included in the current study, the average FD was 0.13 mm (SD = 0.05), the average standardized DVARS was 1.24 (SD = 0.09), and on average, 0.2% (SD = 0.25) of the scans showed spikes across 300 volumes. We further denoised resting-state data using the XCP Engine pipeline (Version 1.0)^38^. Specifically, the following motion-related confounds were removed from BOLD sequences: (1) de-meaning and detrending, (2) de-spiking using AFNI’s 3dDespike utility, (3) bandpass filtering (0.01–0.08 Hz), (4) 36-parameter confound regression including 6 realignment parameters, mean signal in white-matter, cerebrospinal fluid, and mean global signal, as well as the first power and quadratic expansions of their temporal derivatives. Please see [SI1] for additional information on the preprocessing and [SI2] for more details and effectiveness of our motion correction.

#### Frontoparietal functional connectivity

**Figure 1:**
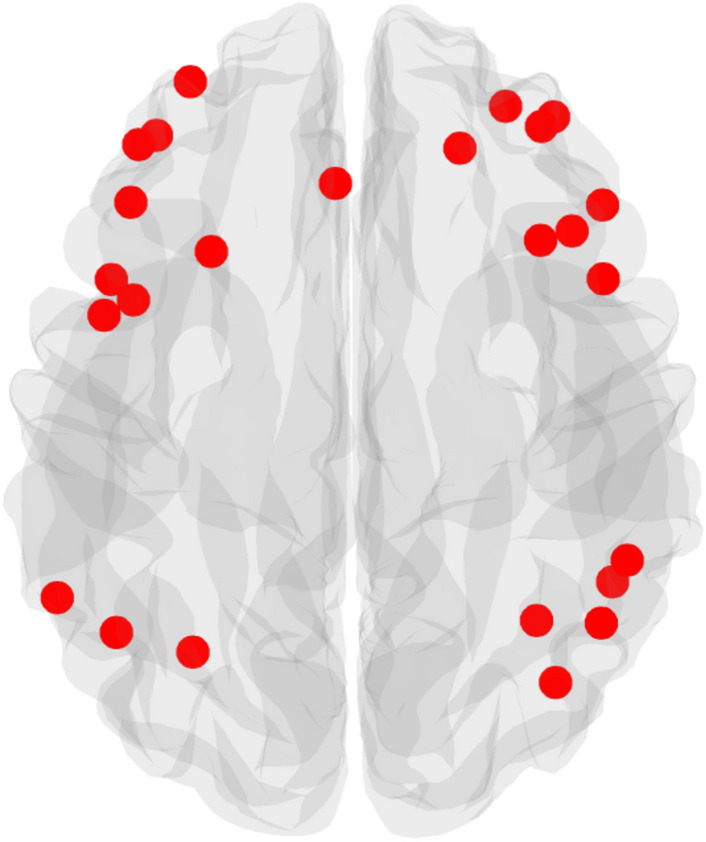
Frontoparietal nodes. The frontoparietal system consisted of 25 frontoparietal nodes defined by Power et al., 2011, with 5 mm diameter spheres around the center coordinate.

The frontoparietal system was defined based on a previously established functional brain parcellation from the Power 264 atlas^39^, which provides a coarse resolution of brain networks. We selected 25 regions or *nodes* that were defined by 5 mm diameter spheres around the center coordinate (Fig. 1, [SI3] for MNI coordinates), that represent the frontoparietal system. We extracted BOLD time series of each node and calculated within-person Pearson’s correlation coefficients between every pair of node time courses. We applied a Fisher *z*-transform to the correlation coefficients within each participant, on a per-participant basis, and focused on positive coefficients given the ambiguity of interpreting negative edges due to regression of the global signal during preprocessing of the rs-fMRI data^40–42^. Available correlation coefficients from edges between 25 nodes within the frontoparietal system were then averaged to indicate a summary index of average frontoparietal functional connectivity per participant. To explore additional levels of granularity, we also examined functional connectivity within subregions of the frontoparietal system to further understand which specific subsystems^43^ were responsible for the hypothesized results [SI4]. In addition, as control measures, we repeated main analyses using functional connectivity within the visual and auditory systems [SI5]. Results from intraclass correlation coefficient (ICC) analyses indicated moderate reliability across the whole brain (ICC = 0.56) and within the frontoparietal system (ICC = 0.52).

### Positionality statement

Mindful that our identities can influence our approach to science, the authors wish to provide the reader with information about our backgrounds. With respect to gender, when the manuscript was drafted, seven authors self-identified as women, four as men, and two as non-binary. With respect to race, nine authors self-identified as White, three as Asian, and one as Black. None of the authors are current college students (i.e., the main population sampled).

### Citation diversity statement

Recent work in several fields has identified a bias in citation practices such that papers from women and other minority scholars are under-cited relative to the number of such papers in the field^44,45^. Here we sought to consider choosing references that reflect the diversity of the field in thought, form of contribution, gender, and other factors. We obtained the predicted gender of the first and last author of each reference by using databases that store the probability of a first name being carried by a woman^46^. By this measure, our references contain 25.18% woman(first)/woman(last), 8.87% man/woman, 25.58% woman/man, and 40.38% man/man. This method is limited in that a) names, pronouns, and social media profiles used to construct the databases may not, in every case, be indicative of gender identity and b) it cannot account for intersex, non-binary, or transgender people.

### Measures

#### Ecological momentary assessment surveys

Throughout the 28-day EMA period, participants received two surveys per day via mobile app in the morning (8AM) and evening (6PM) that assessed their social media use and current affect, described below.

##### Time spent on social media

Participants retroactively reported how much time they spent on social media since the last survey. Participants self-determined what they meant by social media (“Since the previous survey, how much time have you spent on social media?”), and chose one of the following options: 0 = have not checked social media, 1 = less than 10 min, 2 = 10–30 min, 3 = 31–60 min, 4 = 1–2 h, 5 = 2–3 h, 6 = 3–4 h, or 7 = more than 4 h. We then converted the original scores into minutes, by taking the midpoint value of the answer range: 0 = 0 min, 1 = 5 min, 2 = 20 min, 3 = 45 min, 4 = 90 min, 5 = 150 min, 6 = 210 min, 7 = 270 min). All findings remained consistent using raw scores [SI6].

##### Current negative affect

Participants answered four separate questions that assessed their current levels of negative affect (How negative / sad / anxious / angry do you feel right now?) on a scale of 1 (not at all) to 100 (extremely) with higher scores indicating higher negative affect. To ensure appropriate reliability to detect within-person change over time (reliability of change [*R*c])^47^, four items were combined to create mean negative affect scores (*R*c = 0.752). Surveys also included two positive affect items (How positive/happy do you feel right now?), which we combined to produce mean positive affect scores (*R*c = 0.830) [SI7].

#### Self-report surveys

Participants self-reported their levels of depression, anxiety, emotion dysregulation, and demographic information prior to the fMRI scan.

##### Depression

Depressive symptoms were measured by the 10-item Center for Epidemiologic Studies Depression Scale (CES-D)^48^. Items were rated on a 0 (rarely or none of the time) to 3 (most or all the time) scale. Scores were coded such that higher values indicate higher depressive symptom severity, then summed with a score range of 0–30. The scale’s internal consistency in the current study (α = 0.80) was high.

##### Anxiety

The 20-item State-Trait Anxiety Inventory (STAI)^49^ measured participants’ levels of anxiety symptoms. Items were rated on a 1 (not at all) to a 4 (very much) scale. Scores were coded such that higher values indicate higher levels of anxiety, then summed with scores ranging from 20 to 80. The scale’s internal consistency in the current study (α = 0.89) was high.

##### Emotion dysregulation

The short form 18-item Difficulties in Emotion Regulation Scale (DERS)^50,51^ measured the degree of difficulty experienced when regulating emotions. Items were rated on a 1 (almost never) to a 5 (almost always) scale. Scores were coded such that higher values indicate greater difficulty regulating emotions and were then averaged. DERS consists of six subscales measuring different types of difficulty in emotion regulation; we report the overall score in the main text, since our goal was to examine the general levels of emotion dysregulation. Please see [SI8] for individual subscale results. The scale’s internal consistency in the current study (α = 0.90) was high.

##### Demographics

Participants self-reported their age, gender, race/ethnicity, and perceived status within their communities using the MacArthur Scale of Subjective Social Status^52^. The race/ethnicity variable was converted to indicate White, Asian, Black, Latino/a, and Multiracial (i.e., selected more than one race/ethnicity option).

### Analysis plan

We explored five hypotheses using three separate models. First, three linear regression models tested the associations between individual differences in average resting state frontoparietal connectivity and (1) depression (CES-D), (2) anxiety (STAI), and (3) emotion dysregulation (DERS).

Next, we used the “lmer” function of the lme4 package in R (ver.1.1-26)^53^ to perform a multilevel analysis. The model included the amount of time spent on social media, frontoparietal connectivity, and their interaction term as predictors of subsequent negative affect. To focus on within-person relationships, the time-varying predictor variable was within-person standardized. That is, we created a within-person standardized version of the time-spent-on-social-media variable, and each person’s time series had a mean of 0 and a standard deviation of 1. This allowed us to focus on within-person changes (i.e., in comparison to an individuals’ usual level) while holding the between-person differences in time spent on social media constant. To account for potential issues with combining multisite data, we included the site as a second level variable (i.e., participants nested within sites). Using this model, we tested whether (4) longer social media use during the period since the previous time point would subsequently predict more negative affect (EMA measures were obtained concurrently but the temporal association was assumed based on the retroactive wording of the social media use question; we focused on all temporal relationships, including morning-to-evening and evening-to-morning). In the same model, we further explored whether (5) the temporal link between social media use and negative affect would be moderated by individual differences in resting state frontoparietal functional connectivity. The slope of the time spent on social media was allowed to vary randomly across participants.

We then conducted follow-up simple slopes analysis, using the “sim_slopes” function of the interactions package in R (ver.1.1.3)^54^. Simple slopes analysis involves extracting predicted values of the relationship between predictor and outcome variables at different levels of a moderator, using all of the data directly from the multilevel model^55^. Using this method, we explored whether the relationship between social media use and negative affect varied across three different levels of frontoparietal functional connectivity, including at the mean and one standard deviation below/above the group mean level of connectivity. We also explored the possibility that the negative/positive affect at a previous time point would predict later social media use, and whether these links might be moderated by frontoparietal functional connectivity [SI9].

All analysis controlled for the study condition as part of a parent study that is not the focus of the current investigation. Analyses also controlled for demographic variables including age, gender, race/ethnicity, and self-reported social status. The multilevel model initially did not converge, and we removed covariates that accounted for no variance until the model converged, which resulted in the removal of the social status covariate. Simple slopes analysis models failed to converge when participants were nested within sites; the site variable was therefore removed for the simple slopes analysis. All results remained robust without controlling for these potential covariates [SI10]. All main results were robust to false discovery rate correction [SI11]. Unstandardized beta coefficients (b) and 95% confidence intervals (CI) are reported. All reported *p* values are two-tailed. Analyses were performed in R (v3.6.1, www.r-project.org) using the R-studio interface (v1.2.1335). Coefficients and statistics for all models are reported in [SI12].

## Results

### Time spent on social media

Throughout the 28-day EMA period, all participants reported at least some (i.e., non-zero) minutes of social media use. On average, participants reported having used social media on about 23 out of 28 days (*M* = 23.37 days, *SD* = 7.08, median = 26 days, range = 1–28). Participants spent about an hour on social media per use (assessed twice per day; *M* = 60.82 min, *SD* = 52.69 min, median = 44 min, range = 0–270 min), which translated into roughly 2 h of daily social media use. This rate was comparable to the average daily social media use among the U.S. adults (2 h 14 min)^1^.

### Associations between functional connectivity and depression, anxiety, and emotion regulation

**Figure 2:**
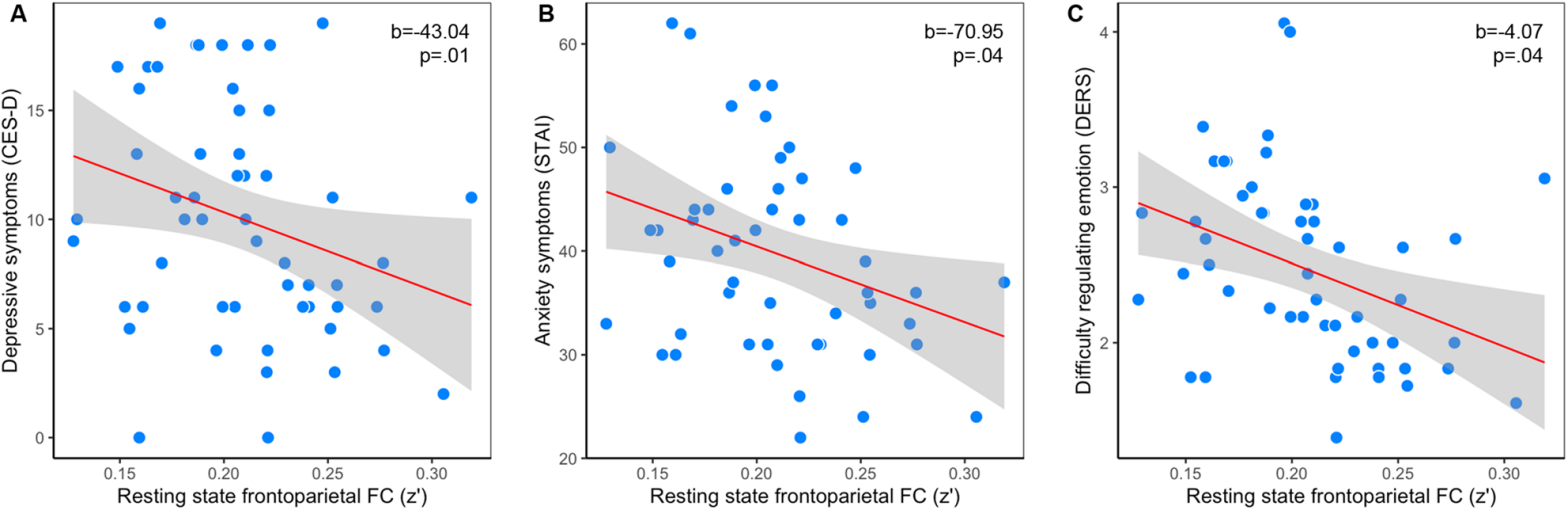
Frontoparietal functional connectivity, depression, anxiety, and difficulty regulating emotion. Stronger resting state functional connectivity within the frontoparietal system was associated with lower self-reported depression (**A**), lower self-reported anxiety (**B**), and greater self-reported difficulty regulating emotions (**C**) among college students. CES-D = Center for Epidemiologic Studies Depression Scale; DERS = Difficulties in Emotion Regulation Scale; FC (z’) = Fisher* r*-to-*z* transformed functional connectivity scores; STAI = State-Trait Anxiety Inventory.

To assess whether individual differences in resting state functional connectivity within the frontoparietal system relate to emotional dysfunction, we examined its relationship to self-reported levels of depression, anxiety, and difficulty in emotion regulation measured at baseline. Participants who showed lower average frontoparietal connectivity also self-reported higher levels of depression (N = 51; b = − 43.043, 95%CI [− 73.05, − 13.03], *p* = 0.006) and anxiety (N = 51; b = − 70.947, 95%CI [− 136.70, − 5.20], *p* = 0.035), as well as greater difficulty regulating emotions (N = 52; b = − 4.069, 95%CI [− 7.86, − 0.28], *p* = 0.036) (Fig. 2).

### Relationships among social media use, functional connectivity, and subsequent negative affect

A multilevel model examined the relationship between social media use and negative affect, and whether this relationship varied by individual differences in resting state frontoparietal functional connectivity (N = 54; 2424 observations). Spending more than one’s usual number of minutes on social media since the previous time point predicted greater increases in negative affect (b = 4.382, 95%CI [1.37, 7.40], *p* = 0.006). In the same model, we also observed that this link was moderated by average frontoparietal functional connectivity, such that greater functional connectivity was associated with a weaker relationship between social media use and negative affect (b = − 17.154, 95%CI [− 31.10, − 3.21], *p* = 0.019).

**Figure 3:**
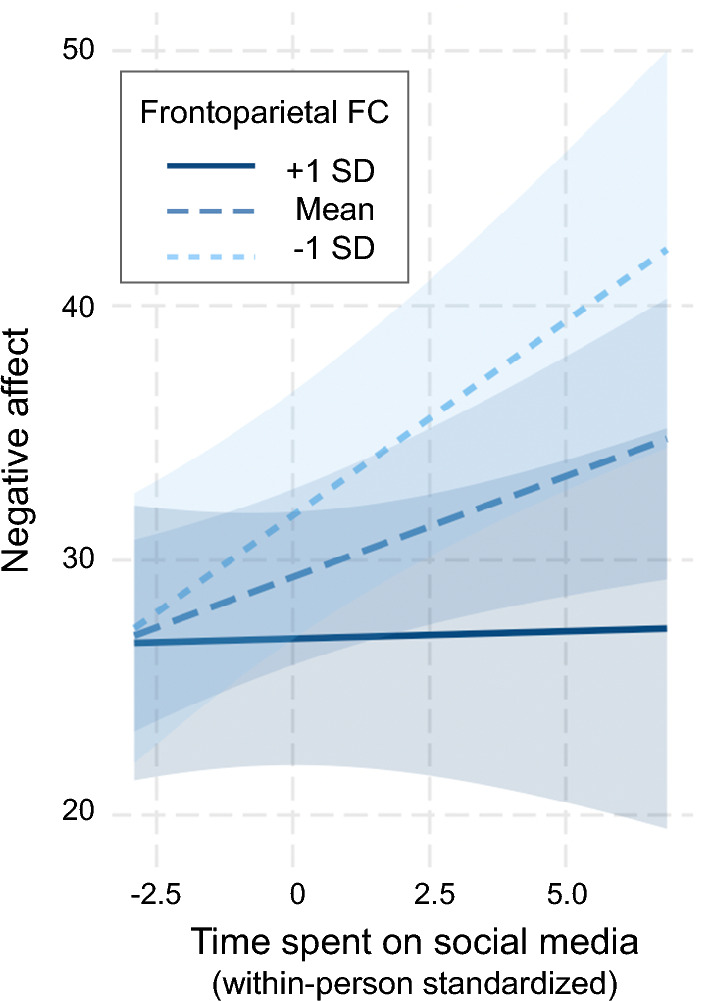
Simple slopes analysis depicting the temporal relationship between time spent on social media and subsequent negative affect. Individuals with the weakest and mean levels of resting state functional connectivity within the frontoparietal system at baseline reported greater increases in negative affect following longer than their usual social media use, using time varying reports collected twice a day. By contrast, time spent on social media was not associated with subsequent negative affect for individuals with stronger frontoparietal functional connectivity. FC = functional connectivity; SD = standard deviation.

Results from follow-up simple slopes analyses showed that more minutes spent on social media predicted feeling more negative for individuals with lower than average (b = 1.551, 95%CI [0.71, 2,39], *p* = 0.001) or at the mean (b = 0.746, 95%CI [0.15, 1.34], *p* = 0.017) levels of frontoparietal functional connectivity. By contrast, time spent on social media was not associated with subsequent negative affect among individuals with higher-than-average levels of frontoparietal functional connectivity (b = − 0.058, 95%CI [− 0.90, 0.78], *p* = 0.892) (Fig. 3).

We also explored the reverse of the shown temporal relationship between social media use and subsequent affect. Specifically, we tested whether feeling positive or negative at a previous time point would predict the number of minutes spent on social media reported at a later time point, and whether this relationship would be moderated by frontoparietal functional connectivity; we found no such relationships [SI9]. In addition, none of the frontoparietal system’s specific subsystems, alone, moderated the link between time spent on social media and negative affect [SI4], suggesting that the interaction effect was unique to the average frontoparietal functional connectivity. We also examined positive affect as an outcome and found no significant effect of minutes spent on social media and subsequent positive affect, or an interaction between time spent on social media and frontoparietal connectivity [SI7].

## Discussion

Social media have become part of many individuals’ daily routines and may affect users’ day-to-day experiences. The past decade of research suggests that the effects of social media engagement likely vary by individual differences that shape the user experience^5^. The current study explored whether individual differences in functional connectivity within the frontoparietal system moderated the relationship between social media use and subsequent affect.

As a potential neural moderator of the relationship between social media experiences and affective experiences, we focused on the average resting state functional connectivity within the frontoparietal system implicated in cognitive control of emotion^12,13^. In our non-clinical college sample, lower average frontoparietal functional connectivity was associated with higher depressive and anxiety symptoms, as well as greater difficulties in regulating emotions. These results suggest that the previously shown link between hypoconnectivity within the frontoparietal system and depression/anxiety^22,23^ may generalize to individuals without major psychopathology. Furthermore, weaker frontoparietal connectivity may signal a risk for emotion dysregulation. Future studies may examine whether changes in frontoparietal connectivity covary with different profiles of negative affect and emotion regulation capacities in a wider range of non-clinical populations to determine its role in everyday affective experience.

Does spending time on social media lead to feeling worse, or does feeling bad lead to longer social media use? Indeed, social media use is a tempting solution for a quick mood fix, and people may seek social media to regulate emotions^56,57^. However, we did not observe any evidence of positive or negative affect preceding longer/shorter social media use [SI9], suggesting that affect might not be a significant predictor of minutes spent on social media. Instead, our data showed that the reverse might be true, where spending longer than one’s typical amount of time on social media may precede feeling worse. Previous studies found that passive, as opposed to active, social media use that involves mindlessly scrolling with low engagement with other users^58^ was associated with negative emotions such as increased depression^59^ and anxiety symptoms^60^. Although we did not assess whether participants were actively or passively engaged with social media, nor what types of social media they engaged with (i.e., participants self-determined what they meant by social media), it is possible that longer use in our data may reflect increases in passive use and/or transitioning from active to passive use. Admittedly, time spent on social media might be too coarse an indication of the patterns of engagement^8,61^, but may still provide a rough guideline for a point of intervention. For example, future studies may leverage existing smartphone functionalities to track and limit screen time (e.g., iPhone's Screen Time function), and identify optimal timing of interventions designed to reduce use.

Weaker average functional connectivity within the frontoparietal system was a risk factor for negative affect following social media use. Specifically, spending longer time on social media predicted subsequently feeling worse for those with weaker frontoparietal functional connectivity. This result suggests that social media might pose greater risk to children and adolescents who show immaturities in the development of frontoparietal connectivity^62^, cognitive control of negative emotions^63^, and overall emotion regulation capacities^64^.

Although our study did not include measures of a domain general cognitive control capacity, results suggest that resting state hypoconnectivity within the frontoparietal system may underlie deficits in cognitive control that support emotion regulation in depression^23^. By contrast, stronger frontoparietal connectivity may signal more efficient engagement of executive control that supports emotion regulation in response to salient social information. If this is the case, then efforts to mitigate potential negative effects of social media use may benefit by focusing on specific individual emotion regulation capacities as well as exposures to daily stressors that may disrupt the ability to regulate emotions^65^; future research is needed to test these possibilities. It is also possible that frontoparietal connectivity may preemptively facilitate the filtering of incoming information^9^, selectively engaging with the types of information that are less likely to worsen emotions, and/or preventing rumination or worries when triggered. Given the constantly shifting nature of information processing in the social media environment, these types of regulation likely occur spontaneously. Future studies may examine whether consciously altering frontoparietal activity, via mindfulness^66^ or reappraisal^10^ strategies, may help improve the affective outcomes of social media use.

We note several limitations of this study. First, we relied on self-reported minutes of social media use, which did not distinguish different types of use and may suffer from recall bias and moderate reliability^67^. We also relied on a single-item measure of social media use^8^, which may result in overestimation among light users and underestimation among heavy users^68^. Our use of longitudinal EMA methods that assessed social media use at multiple timepoints per day might have circumvented some of these recall concerns, but does not tell us what people were doing in their use. Therefore, objectively logged and/or more fine-grained measures of the type of social media use will be important to refine these findings.

Second, the EMA survey was conducted at the onset of the COVID-19 pandemic, when there was a surge of negative news on social media (though we note that negative content such as polarizing news^69^ has been endemic in social media environments outside the pandemic context). We also note that in the same dataset, consuming specifically COVID-related news did not significantly interact with frontoparietal connectivity to predict negative affect (b = − 16.056, 95%CI [− 33.64, 1.53], *p* = 0.080). Future studies may test the generalizability of the current findings in additional contexts.

Third, we mainly focused on the *within*-level functional connectivity of the frontoparietal system. However, the frontoparietal system is closely interconnected with other systems (e.g., default mode and attention systems^17^), and may be influenced by the functioning of other systems (e.g., salience system^70^). Given that the frontoparietal recruitment likely involves dynamic configurations between networks across the brain more broadly, it is possible that between network dynamics, or functional coupling between systems, in addition to variability within the frontoparietal system, could play important roles in the context of cognitive control. Furthermore, frontoparietal systems are defined differently across different parcellations with potential overlap across different definitions of the granular functions of control they support. In this initial investigation of the relationship between social media use, which is a broad class of activities, and emotional experience, we began with a commonly used atlas that balances trade-offs between broad network definitions and more granular subsystem specificity. Given that there were statistical interactions between the frontoparietal system as defined in this study and social media use on subsequent affect, future research that is more granular both in terms of the bounds of what types of social media use, and the specific subsystems or patterns of connectivity within and between systems^16,71^, could be helpful^16,71^.

Fourth, psychological processes underlying frontoparietal connectivity should be interpreted with careful considerations for individual- and symptom-specific contexts. Although frontoparietal hypoconnectivity has been found in depression, suggesting reduced communication among neural systems involved in emotion regulation^23^, strong connectivity may not always reflect adaptive emotion processing. For example, over-recruitment of the frontoparietal–posterior cingulate cortex–precuneus system has been associated with cognitive anxiety, suggesting allocation of working memory and attention systems to potential sources of threat^72,73^. In fact, rather than hyper- or hypo-functional connectivity, the flexibility of frontoparietal functional connectivity might be important to interact with the ever-changing social media environments. Emotion regulation flexibility, or the ability to regulate emotions in ways that recognize and adapt to situational demands^74^, has been proposed to be the key to healthy psychological functioning^75,76^. Similarly, being able to efficiently recruit nodes of the frontoparietal system when necessary may be key to regulating negative emotions, which our data cannot address since our study did not include tasks that explicitly required regulation of negative affect. Researchers may combine and compare frontoparietal functioning at rest and while actively regulating emotions^77^, and link the brain data to the degree of emotion regulation success.

Fifth, we acquired relatively short, 5 min of resting state fMRI data. Although some previous studies showed that the strength of functional connectivity was stable with this amount of data^78^, and our use of relatively fast sampling rate (1 s TR) and multiband acquisition likely benefited the short resting state fMRI scan^79^, we encourage future studies on this topic to acquire longer resting state data to improve reliability. Sixth, we note that the current study was a multisite study that involved data collection from two separate universities. Although we used the same scanner model and the same acquisition protocol across sites and modeled participants as nested within sites in our multilevel model where possible, combining multisite fMRI data can introduce site-related confounds^80^. Finally, previous studies support that social media use can increase both positive and negative health outcomes, and depends on the type of engagement^8^. Although we did not observe any associations between time spent on social media and positive affect [SI7], the link between social media use and negative affect that we observed in the current study does not preclude that other social benefits may exist.

In conclusion, this study provides initial evidence that individual differences in average resting state functional connectivity within the frontoparietal system moderate the relationship between time spent on social media and subsequent negative affect. These results are consistent with previous neurocognitive models of negative emotions and emotion regulation in which disrupted communication within the nodes of functional networks may relate to dysregulation of negative emotional experience. More broadly, the current findings suggest potential venues for interventions that consider individual differences in how people respond to social media and the temporal dynamics of social media use that may influence psychological well-being.

### Supplementary Information

Supplementary Information.

## Data Availability

Data and analysis scripts are available at https://github.com/cnlab/social_media_brain/.
