# Supplementary material for: Frontoparietal functional connectivity moderates the link between time spent on social media and subsequent negative affect in daily life
Source: Sci Rep. 2023 Nov 22;13:20501. doi: 10.1038/s41598-023-46040-z (PMC10665348; doi:10.1038/s41598-023-46040-z)
Supplement: Supplementary file 1 — Supplementary Information. [file 41598_2023_46040_MOESM1_ESM.pdf]

**Supplementary Information [SI] for**

# Frontoparietal functional connectivity moderates the link between time spent on social media and subsequent negative affect in daily life

Yoona Kang*, Jeesung Ahn, Danielle Cosme, Laetitia Mwilambwe-tshilobo, Amanda McGowan, Dale Zhou, Zachary M. Boyd, Mia Jovanova, Ovidia Stanoi, Peter J. Mucha, Kevin Ochsner, Dani S. Bassett, David Lydon-Staley & Emily B. Falk*

*Corresponding authors
yoona.kang@rutgers.edu, emily.falk@asc.upenn.edu

Notes: Data and analysis scripts are available in https://github.com/cnlab/social_media_brain. In all multilevel models described in SI, except for the ones presented in SI12, models failed to converge when participants were nested within sites; the site was removed as a second level variable for these analyses.

## SI1. Structural image preprocessing

The T1-weighted (T1w) images were processed using fMRIPrep (Version 20.0.6)[1] based on Nipype 1.4.2[2,3], corrected for intensity non-uniformity (INU) with N4BiasFieldCorrection[4], distributed with ANTs 2.2.0[5], and used as T1w-reference throughout the workflow. The T1w-reference was skull-stripped with a Nipype implementation of the ANTs brain extraction workflow, using OASIS30ANTs as target template. We performed brain tissue segmentation of cerebrospinal fluid, white-matter, and gray-matter on the brain-extracted T1w using fast (FSL 5.0.9)[6]. We reconstructed brain surfaces using recon-all (FreeSurfer 6.0.1)[7] and refined the brain mask estimated previously with a custom variation of the method to reconcile ANTs-derived and FreeSurfer-derived segmentations of the cortical gray-matter of Mindboggle[8]. We performed volume-based spatial normalization to one standard space (MNI152NLin2009cAsym)[9] through nonlinear registration with antsRegistration (ANTs 2.2.0), using brain-extracted versions of T1w reference and the T1w template.

The resting-state fMRI images were processed using the following steps. A reference volume and its skull-stripped version were generated using a fMRIPrep methodology. We estimated a B0-nonuniformity map (or fieldmap) based on two EPI references with opposing phase-encoding directions, with 3dQwarp with AFNI 20160207. Based on the estimated susceptibility distortion, we calculated a corrected EPI reference for a more accurate co-registration with the anatomical reference. We co-registered the BOLD reference to the T1w reference using bbregister from FreeSurfer, which implements boundary-based registration[10]. Co-registration was configured with six degrees of freedom. We estimated head motion parameters with respect to the BOLD reference (transformation matrices, and six corresponding rotation and translation parameters) before any spatiotemporal filtering using mcflirt (FSL 5.0.9)[11]. We slice-time corrected BOLD runs using 3dTshift from AFNI 20160207[12]. We resampled the BOLD time series onto their original native space by applying a single, composite transform to correct for

head motion and susceptibility distortions. Next, the BOLD time series were resampled again into standard space, generating a preprocessed BOLD run in MNI152NLin2009cAsym space. We performed all resamplings with a single interpolation step by composing pertinent transformations (i.e., head motion transform matrices, susceptibility distortion correction when available, and co-registrations to anatomical and output spaces). Gridded (volumetric) resamplings were performed using antsApplyTransforms (ANTs), configured with Lanczos interpolation to minimize the smoothing effects of other kernels[13]. Non-gridded (surface) resamplings were performed using mri_vol2surf (FreeSurfer).

**SI2. Head motion correction**

We estimated head motion parameters with respect to the BOLD reference (transformation matrices, and six corresponding rotation and translation parameters) before any spatiotemporal filtering using mcflirt (FSL 5.0.9)[6]. We slice-time corrected BOLD runs using 3dTshift from AFNI 20160207[12]. We resampled the BOLD time series onto their original native space by applying a single, composite transform to correct for head motion and susceptibility distortions. Next, the BOLD time series were resampled again into standard space, generating a preprocessed BOLD run in MNI152NLin2009cAsym space. We performed all resamplings with a single interpolation step by composing pertinent transformations (i.e., head motion transform matrices, susceptibility distortion correction when available, and co-registrations to anatomical and output spaces). Gridded (volumetric) resamplings were performed using antsApplyTransforms (ANTs), configured with Lanczos interpolation to minimize the smoothing effects of other kernels[13]. Non-gridded (surface) resamplings were performed using mri_vol2surf (FreeSurfer). We calculated various confounds (e.g., framewise displacement [FD], DVARS, global signal) for each TR and logged in a confounds file (for additional details, see https://fmriprep.org/en/20.0.6/outputs.html#confounds). Across the 300 total volumes of the participants included in the current study, the average FD was 0.13mm (SD= 0.05), the average standardized DVARS was 1.24 (SD=0.09), and on average, 0.2% (SD=0.25) of the scans showed spikes across 300 volumes (Fig.SI2a). We manually quality-checked the fMRIPrep outputs, by looking for gross distortions, to ensure adequate preprocessing. None were excluded based on manual checks.

We further denoised resting-state data using the XCP Engine pipeline (Version 1.0)[14]. Specifically, we used XCP Engine to remove motion-related confounds from BOLD sequences: (1) de-meaning and detrending (removal of linear and quadratic trends from time series), (2) de-spiking using AFNI's 3dDespike utility, (3) temporal bandpass filtering using a first-order Butterworth filter to retain signal in the range 0.01-0.08 Hz, (4) 36-parameter confound regression including 6 realignment parameters, mean signal in white-matter, cerebrospinal fluid, and mean global signal, as well as the first power and quadratic expansions of their temporal derivatives. Instead of removing volumes identified as motion outliers, we denoised BOLD time-series voxel-wise by truncating large spikes. None of the participants were entirely excluded for excessive head motion. Participants' resting state frontoparietal functional connectivity levels were not associated with head motion descriptors including FD (r=-0.153, $p$=0.266), DVAR (r=-0.094, $p$=0.501), or percentage of scans that showed spikes (r=0.072, $p$=0.603). Please see

Fig.SI2b for temporal signal to noise ratio (tSNR) maps which were computed by taking the mean signal over time divided by the standard deviation over time.

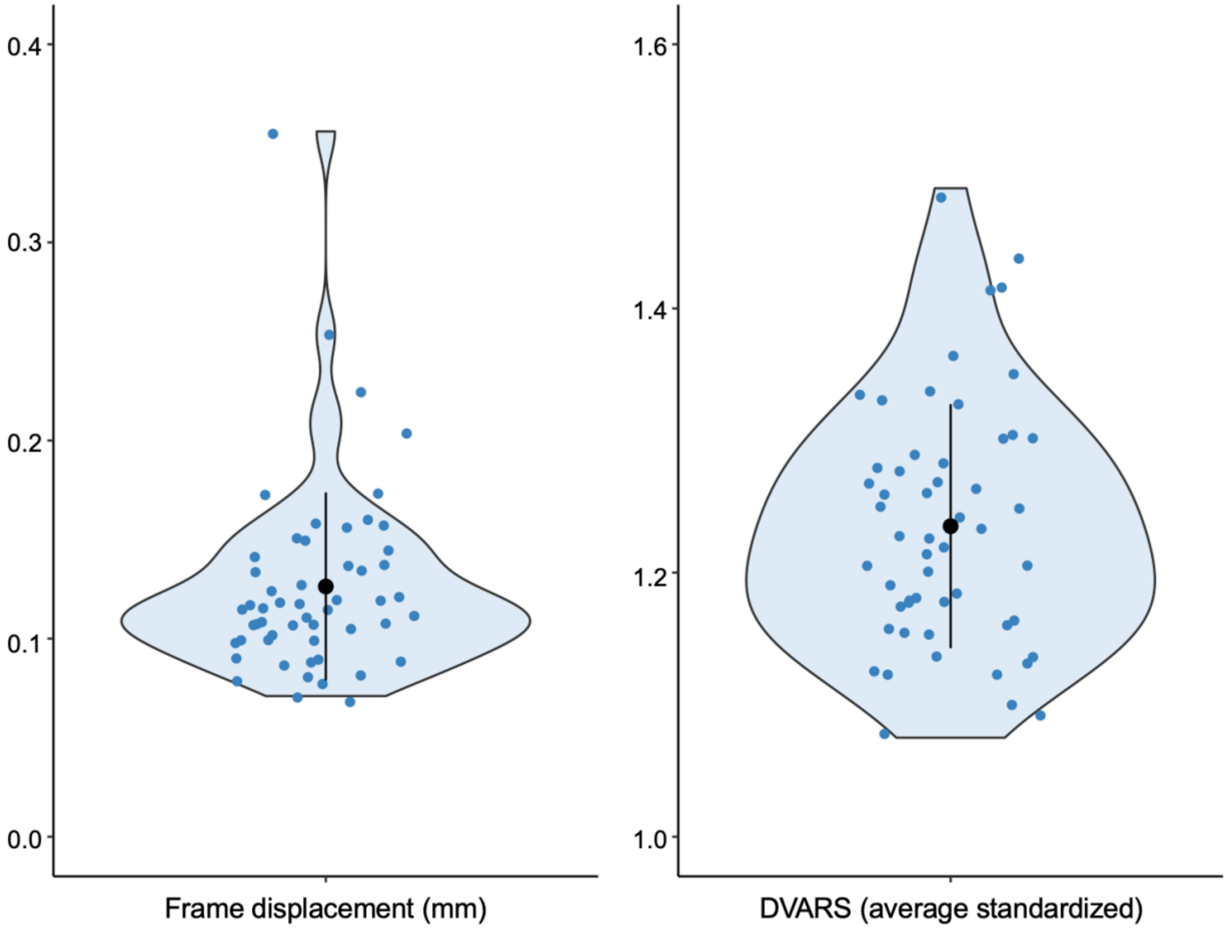


**Fig.SI2a Distributions of motion metrics**

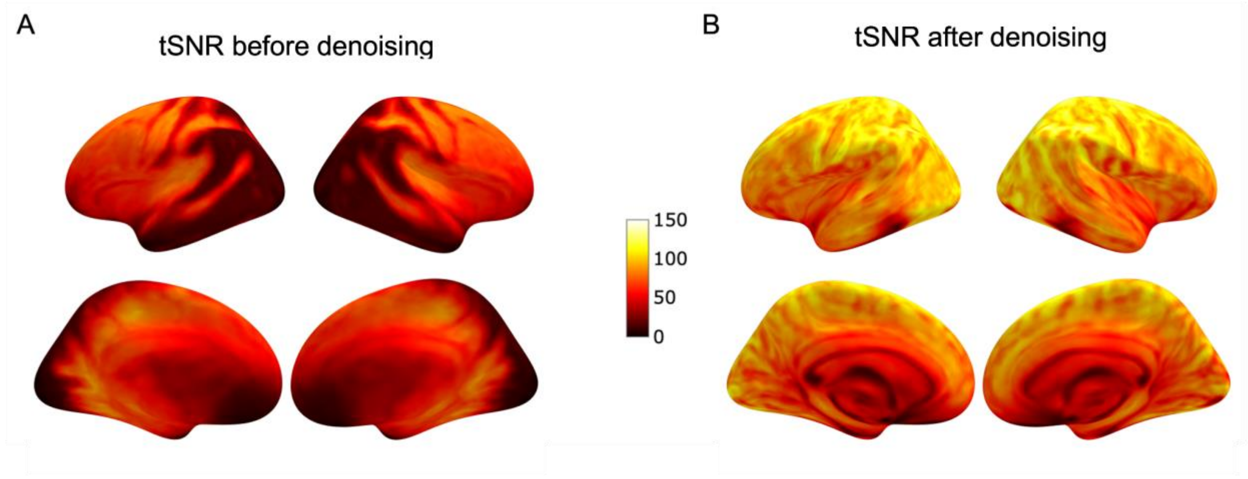


**Fig.SI2b Temporal Signal to Noise Ratio (tSNR) maps**
tSNR maps were constructed using the (A) raw resting-state data and (B) denoised resting-state data. For each map, we averaged across participants and projected the distribution of tSNR across the cortex onto an inflated fsaverage brain surface. Results indicate overall improvement in signal quality, particularly in the occipital and parietal cortices, as well as areas prone to signal dropout such as the temporal lobes and the orbitofrontal cortices.

**SI3. MNI coordinates and corresponding regions of frontoparietal nodes.**
Nodes making up the frontoparietal system were based on the original regions of interest from Power et al., 2011[15]. Regions were determined using the Talairach Daemon[16]. L = left hemisphere, R = right hemisphere.

| **MNI Coordinates** | | | **L/R** | **Region** |
|---|---|---|---|---|
| **X** | **Y** | **Z** | | |
| -44 | 2 | 46 | L | Precentral Gyrus |
| 48 | 25 | 27 | R | Inferior Frontal Gyrus (pars Triangularis) |
| -47 | 11 | 23 | L | Inferior Frontal Gyrus (pars Triangularis) |
| -53 | -49 | 43 | L | Inferior Parietal Lobule |
| -23 | 11 | 64 | L | Middle Frontal Gyrus |
| 58 | -53 | -14 | R | Inferior Temporal Gyrus |
| 24 | 45 | -15 | R | Superior Frontal Gyrus (pars Orbitalis) |
| 34 | 54 | -13 | R | Superior Frontal Gyrus (pars Orbitalis) |
| 47 | 10 | 33 | R | Precentral Gyrus |
| -41 | 6 | 33 | L | Precentral Gyrus |
| -42 | 38 | 21 | L | Middle Frontal Gyrus |
| 38 | 43 | 15 | R | Middle Frontal Gyrus |

| | | | | |
|---|---|---|---|---|
| 49 | -42 | 45 | R | Inferior Parietal Lobule |
| -28 | -58 | 48 | L | Inferior Parietal Lobule |
| 44 | -53 | 47 | R | Inferior Parietal Lobule |
| 32 | 14 | 56 | R | Middle Frontal Gyrus |
| 37 | -65 | 40 | R | Angular Gyrus |
| -42 | -55 | 45 | L | Inferior Parietal Lobule |
| 40 | 18 | 40 | R | Middle Frontal Gyrus |
| -34 | 55 | 4 | L | Middle Frontal Gyrus |
| -42 | 45 | -2 | L | Middle Frontal Gyrus (pars Orbitalis) |
| 33 | -53 | 44 | R | Angular Gyrus |
| 43 | 49 | -2 | R | Middle Frontal Gyrus (pars Orbitalis) |
| -42 | 25 | 30 | L | Inferior Frontal Gyrus (pars Triangularis) |
| -3 | 26 | 44 | L | Superior Medial Frontal Gyrus |

**SI4. Subregions within the frontoparietal system**

Subsystems of the frontoparietal system are implicated in different types of executive control. Specifically, frontoparietal network A ($FP_A$) connects to the default mode network and supports introspective processes, whereas $FP_B$ connects to the dorsal attention network and helps regulate perceptual attention[17]. To further understand which subsystems are more responsible for moderating the link between social media use and affect, we extracted average resting state functional connectivity within these subsystems separately using Schaefer 17 systems atlas with 400 parcels[18,19].

We ran two separate multilevel models that included the amount of time spent on social media, $FP_A$ / $FP_B$ subsystems within the frontoparietal system (in separate models), and their interaction term as predictors of subsequent negative affect. We found that the main interaction we observed in the main text (frontoparietal functional connectivity x time spent on social media predicting negative affect) was unique to overall average frontoparietal connectivity, but not to the specific subsystems: We did not find any significant interaction between time spent on social media and $FP_A$ (b=-1.028, 95% CI [-11.96, 9.90], t=-0.184, *p*=0.854) or $FA_B$ (b=-6.335, 95% CI [-18.46, 5.79], t=-1.025, *p*=0.310). These results suggest that the overall executive control, rather than its subdomains, might be more important for regulating complex social stimuli such as the ones presented in social media.

**SI5. Functional connectivity within visual and auditory systems as control measures**

As one test of specificity, we repeated all main analyses using functional connectivity within the visual and auditory systems[15]. We chose these regions because, compared to the frontoparietal system, we had less theoretical reason to believe they are involved in cognitive control, emotion regulation, or negative affect. Among the additional regions examined, we found that the results reported in the main text were unique to the frontoparietal system. Specifically, individual

differences in functional connectivity within the visual and auditory systems were not significantly associated with the levels of depression (visual: b=3.969, *p*=0.552; auditory: b=-8.516, *p*=0.242), anxiety (visual: b=7.845, *p*=0.577; auditory: b=-3.771, *p*=0.807), or difficulty regulating emotions (visual: b=0.089, *p*=0.914; auditory: b=-1.618, *p*=0.065). In addition, visual/auditory functional connectivity did not moderate the relationship between minutes spent on social media and negative affect (visual: b=3.921, *p*=0.227; auditory: b=-5.440, *p*=0.138).

**SI6. Results using raw scores for the time spent on social media measure**
Twice a day, participants received ecological momentary assessment (EMA) surveys that asked about the amount of time they spent on social media since the last survey ("Since the previous survey, how much time have you spent on social media?") by choosing one of the following options: 0=have not checked social media, 1=less than 10 mins, 2=10–30 mins, 3=1–60 mins, 4=1–2 hrs, 5=2-3 hrs, 6=3-4 hrs, or 7=more than 4 hrs. In the main text, we report results that converted these raw scores into minutes by taking the midpoint value of the answer range. All findings remained consistent when we used the raw scores: More minutes spent on social media since the previous time point predicted greater increases in negative mood (b=4.581, 95% CI [1.52, 7.64], t=2.932, *p*=0.005). In the same model, we also observed that this link was moderated by frontoparietal functional connectivity, such that greater functional connectivity was associated with a weaker relationship between social media use and negative affect (b=-18.953, 95% CI [-33.32, -4.59], t=-2.586, *p*=0.013).

**SI7. Current positive affect**
In addition to the negative EMA questions reported in the main text, participants also reported their current positive affect by responding to two positive affect items, twice a day (How positive / happy do you feel right now?) on a scale of 1 (not at all) to 100 (extremely) with higher scores indicating higher positive affect. We combined these two ratings to produce mean positive affect scores ($R$c =0.830). Using these positive affect composite scores, we tested the relationships between time spent on social media, frontoparietal functional connectivity, and positive affect. We did not find any association between time spent on social media reported since the previous time point and current positive affect (b=-1.813, 95% CI [-6.16, 2.54], t=-0.817, *p*=0.418). In the same model, we also did not find any significant interaction between time spent on social media and frontoparietal functional connectivity in predicting positive affect (b=8.586, 95% CI [-11.83, 29.01], t=0.824, *p*=.414).

**SI8. Difficulties in Emotion Regulation (DERS) subscales**
The short form 18-item Difficulties in Emotion Regulation Scale (DERS)[20,21] measures the degree of difficulty experienced when regulating emotions, rated on a 1 (almost never) to a 5 (almost always) scale. DERS includes six subscales that measure different types of difficulties in emotion regulation, including lack of emotional awareness, nonacceptance of emotions, impulse control difficulties, restricted access to emotion regulation strategies, reduced emotional clarity, and difficulties participating in goal-directed behavior. In the main text, we report that the greater emotion regulation dysfunction, indicated by higher DERS composite scores, was

associated with weaker frontoparietal functional connectivity. Here, we examined this relationship across different subscales within DERS. Correlation analysis showed that, of the six DERS subscales, two subscales likely drove the inverse relationship between the frontoparietal functional connectivity (FC, in the figure below) and emotion dysregulation, including goal-directed behavior (*r*=-.394 95% CI [-0,60, -0.14], *p*=0.003) and restricted access to emotion regulation strategies (*r*=-.422, 95% CI [-0,62, -0.17], *p*=0.002). These results are consistent with previous evidence supporting the role of the frontoparietal system in engagement of executive control that support emotion regulation, such as goal-directed cognition and strategic planning[22–25].

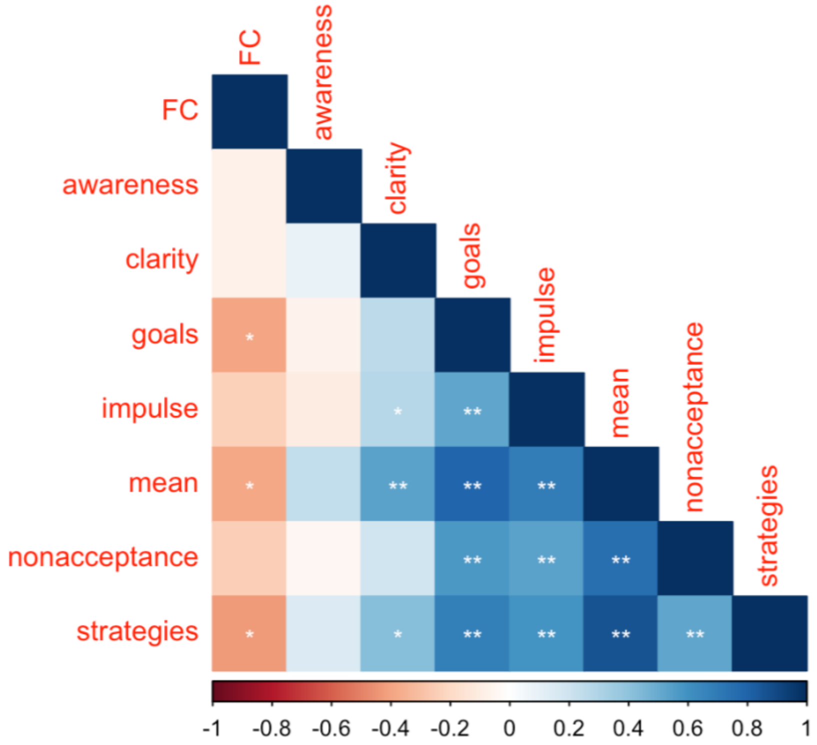


**Fig.SI8 Bivariate correlations between frontoparietal functional connectivity and Difficulties in Emotion Regulation (DERS) subscales**

Notes: FC=Functional Connectivity within the frontoparietal system, mean=average DERS score

**SI9. Temporal relationships between time spent on social media and affect**

To further unpack the temporal link between social media use and subsequent affect, we also explored the possibility that the negative or positive affect at a previous time point would predict later social media use, and whether these links might be moderated by frontoparietal functional connectivity. Two multilevel models included the negative or positive affect scores at a previous time point (in two separate models), frontoparietal connectivity, and their interaction term as predictors of subsequent reported time spent on social media. The slope of the positive affect

scores was allowed to vary randomly across participants; the random effect variance of the negative affect was close to zero and was removed from the model. We found no evidence linking previous negative (b=0.187, 95% CI [-0.04, 0.41], t=1.640, *p*=0.101) or positive affect (b=0.012, 95% CI [-0.274, 0.297], t=0.080, *p*=0.936) and the subsequent amount of time spent on social media. We also did not find any interaction between the resting state frontoparietal functional connectivity and negative (b=-0.712, 95% CI [-1.776, 0.34], t=-1.328, *p*=0.184) or positive affect (b=-0.121, 95% CI [-1.457, 1.214], t=-0.178, *p*=0.859) predicting subsequent time spent on social media.

**SI10. Potential covariates and confounds**

We report results including potential covariates in the main text. Here, we repeated the analyses without controlling for potential covariates, including the demographic variables (age, gender, race/ ethnicity, and perceived social status within a campus group) and the condition assignment as part of a larger study. All results remained consistent: Lower frontoparietal connectivity was associated with higher self-reported depression (b=-40.831, 95% CI [-72.20, -9.47], t=-2.615, *p*=0.012), higher self-reported anxiety (b=-72.904, 95% CI [-132.86, -12.95], t=-2.422, *p*=0.018), and greater difficulty regulating emotions (b=-5.355, 95% CI [-9.02, -1.69], t=-2.936, *p*=0.005). Also consistent with the results reported in the main text, more minutes spent on social media since the previous time point predicted greater than usual negative affect (b=4.408, 95% CI [1.40, 7.41], t=2.876, *p*=0.006). In the same model, we also observed that this link was moderated by frontoparietal functional connectivity, such that greater functional connectivity was associated with a weaker relationship between social media use and negative affect (b=-17.241, 95% CI [-31.27, -3.21], t=-2.409, *p*=0.019).

**SI11. Correction for multiple comparisons**

All main results were robust to False Discovery Rate (FDR) correction (FDR-adjusted p values for the association between frontoparietal functional connectivity and depression (0.018), anxiety (0.036), and emotion dysregulation (0.036); for the link between social media and negative affect at lower (0.003), mean (0.026), and higher (0.892) levels of frontoparietal functional connectivity).

**SI12. Coefficients and statistics of all main analyses models.**

| | **β** | **b** | **se** | **95% CI** | **t** | **p** |
|---|---|---|---|---|---|---|
| FC → Depression | -0.363 | -43.043 | 14.836 | -73.05, -13.03 | -2.901 | 0.006 |
| FC → Anxiety | -0.318 | -70.947 | 32.505 | -136.70, -5.20 | -2.183 | 0.035 |

| | | | | | | |
|---|---|---|---|---|---|---|
| FC → Emotion dysregulation | -0.290 | -4.069 | 1.877 | -7.86, -0.28 | -2.168 | 0.036 |
| Social media use → Negative affect | 0.235 | 4.382 | 1.538 | 1.37, 7.40 | 2.849 | 0.006 |
| FC * Social media use → Negative affect | -0.197 | -17.154 | 7.150 | -31.10, -3.21 | -2.411 | 0.019 |

Notes: Standardized (β) and unstandardized (b) regression coefficients, standard error for unstandardized regression coefficients (se), and 95% confidence intervals (CI) are displayed. FC=Functional Connectivity within the frontoparietal system, Depression=Epidemiologic Studies Depression Scale (CES-D), Anxiety=State-Trait Anxiety Inventory (STAI), Emotion dysregulation=Difficulties in Emotion Regulation Scale, social media use=Minutes spent on social media. Time-varying variables (social media use, negative affect) were within-person standardized (N=54; 2424 observations). All analyses controlled for potential covariates, including demographic variables (age, gender, and race/ethnicity), the condition assignment as part of a parent study, and perceived social status (in analyses linking FC and depression, anxiety, and emotion dysregulation). Please see https://github.com/cnlab/social_media_brain/ for the complete model output statistics.

**SI References**


1. Esteban, O. *et al.* fMRIPrep: a robust preprocessing pipeline for functional MRI. *Nat. Methods* **16**, 111–116 (2019).
2. Gorgolewski, K. *et al.* Nipype: a flexible, lightweight and extensible neuroimaging data processing framework in python. *Front. Neuroinform.* **5**, 13 (2011).
3. Gorgolewski, K. J. *et al.* Nipype. *Software. Zenodo. https://doi. org/10. 5281/zenodo* **596855**, (2018).
4. Tustison, N. J. *et al.* N4ITK: improved N3 bias correction. *IEEE Trans. Med. Imaging* **29**, 1310–1320 (2010).
5. Avants, B. B., Epstein, C. L., Grossman, M. & Gee, J. C. Symmetric diffeomorphic image registration with cross-correlation: evaluating automated labeling of elderly and neurodegenerative brain. *Med. Image Anal.* **12**, 26–41 (2008).
6. Jenkinson, M., Bannister, P., Brady, M. & Smith, S. Improved optimization for the robust and accurate linear registration and motion correction of brain images. *Neuroimage* **17**, 825–841 (2002).
7. Dale, A. M., Fischl, B. & Sereno, M. I. Cortical surface-based analysis. I. Segmentation and surface reconstruction. *Neuroimage* **9**, 179–194 (1999).
8. Klein, A. *et al.* Mindboggling morphometry of human brains. *PLoS Comput. Biol.* **13**, e1005350 (2017).
9. Fonov, V. S., Evans, A. C., McKinstry, R. C., Almli, C. R. & Collins, D. L. Unbiased nonlinear average age-appropriate brain templates from birth to adulthood. *Neuroimage* **Supplement 1**, S102 (2009).
10. Greve, D. N. & Fischl, B. Accurate and robust brain image alignment using boundary-based registration. *Neuroimage* **48**, 63–72 (2009).
11. Zhang, Y., Brady, M. & Smith, S. Segmentation of brain MR images through a hidden Markov random field model and the expectation-maximization algorithm. *IEEE Trans. Med. Imaging* **20**, 45–57 (2001).
12. Cox, R. W. & Hyde, J. S. Software tools for analysis and visualization of fMRI data. *NMR in Biomedicine* vol. 10 171–178 Preprint at https://doi.org/10.1002/(sici)1099-1492(199706/08)10:4/5<171::aid-nbm453>3.0.co;2-l (1997).
13. Lanczos, C. Evaluation of Noisy Data. *Journal of the Society for Industrial and Applied Mathematics Series B Numerical Analysis* **1**, 76–85 (1964).
14. Ciric, R. *et al.* Benchmarking of participant-level confound regression strategies for the control of motion artifact in studies of functional connectivity. *Neuroimage* **154**, 174–187 (2017).
15. Power, J. D. *et al.* Functional network organization of the human brain. *Neuron* **72**, 665–678 (2011).
16. Lancaster, J., Kochunov, P., Nickerson, D. & Fox, P. Stand-alone Java-based version of the Talairach daemon database system. *Neuroimage* **5 Supplement**, S923 (2000).
17. Dixon, M. L. *et al.* Heterogeneity within the frontoparietal control network and its relationship to the default and dorsal attention networks. *Proc. Natl. Acad. Sci. U. S. A.* **115**, E1598–E1607 (2018).
18. Schaefer, A. *et al.* Local-Global Parcellation of the Human Cerebral Cortex from Intrinsic

Functional Connectivity MRI. *Cereb. Cortex* **28**, 3095–3114 (2018).
19. Thomas Yeo, B. T. *et al.* The organization of the human cerebral cortex estimated by intrinsic functional connectivity. *J. Neurophysiol.* (2011) doi:10.1152/jn.00338.2011.
20. Gratz, K. L. & Roemer, L. Multidimensional assessment of emotion regulation and dysregulation: Development, factor structure, and initial validation of the difficulties in emotion regulation scale. *J. Psychopathol. Behav. Assess.* **26**, 41–54 (2004).
21. Victor, S. E. & Klonsky, E. D. Validation of a brief version of the difficulties in emotion regulation scale (DERS-18) in five samples. *J. Psychopathol. Behav. Assess.* **38**, 582–589 (2016).
22. Kober, H. *et al.* Functional grouping and cortical–subcortical interactions in emotion: A meta-analysis of neuroimaging studies. *Neuroimage* **42**, 998–1031 (2008).
23. Lindquist, K. A. & Barrett, L. F. A functional architecture of the human brain: emerging insights from the science of emotion. *Trends Cogn. Sci.* **16**, 533–540 (2012).
24. Rodman, A. M., Jenness, J. L., Weissman, D. G., Pine, D. S. & McLaughlin, K. A. Neurobiological Markers of Resilience to Depression Following Childhood Maltreatment: The Role of Neural Circuits Supporting the Cognitive Control of Emotion. *Biol. Psychiatry* **86**, 464–473 (2019).
25. Wessing, I. *et al.* Cognitive emotion regulation in children: Reappraisal of emotional faces modulates neural source activity in a frontoparietal network. *Dev. Cogn. Neurosci.* **13**, 1–10 (2015).
